# Effects of aquatic exercise on mental health, functional autonomy and oxidative stress in depressed elderly individuals: A randomized clinical trial

**DOI:** 10.6061/clinics/2019/e322

**Published:** 2019-06-20

**Authors:** Luciano Acordi da Silva, Luana Tortelli, Janaina Motta, Lorhan Menguer, Sindianra Mariano, Gladson Tasca, Gustavo de Bem Silveira, Ricardo Aurino Pinho, Paulo Cesar Lock Silveira

**Affiliations:** I Laboratorio de Fisiologia e Bioquimica do Exercicio, Grupo de Pesquisa de Exercicios Aquaticos Avancados, Universidade do Extremo Sul Catarinense, Criciuma, SC, BR; II Escola Superior de Criciuma (ESUCRI), Criciuma, SC, BR; III Centro Universitario Barriga Verde (UNIBAVE), Orleans, SC, BR; IV Laboratorio de Fisiopatologia Experimental, Programa de Pos-Graduacao em Ciencias da Saude, Universidade do Extremo Sul Catarinense, Criciuma, SC, BR; V Laboratorio de Bioquimica do Exercicio em Saude, Faculdade de Medicina, Programa de Pos-Graduacao em Ciencias da Saude, Pontifícia Universidade Catolica do Parana (PUCPR), Curitiba, PR, BR

**Keywords:** Depression, Anxiety, Oxidative Stress, Aquatic Exercise

## Abstract

**OBJECTIVES:**

The aim of this study was to investigate the effects of aquatic exercise on mental health, functional autonomy and oxidative stress parameters in depressed elderly individuals.

**METHODS:**

Initially, ninety-two elderly individuals were included in the study and were allocated into the depression group (n=16) and nondepression group (n=14). Both groups engaged in the aquatic exercise program for 12 weeks, including two weekly sessions (45 min/session) at a low intensity (between 50% and 60% of maximal heart rate or Borg scale scores of 13 to 14) throughout the intervention. All outcomes were evaluated at baseline and 12 weeks later.

**RESULTS:**

The patients were 63.5±8.8 years old. The following scores were decreased after training in the depressed group: depression (53%), anxiety (48%), and Timed Up & Go (33%). The following scores increased: Berg Balance Scale (9%) and flexibility (44%). Regarding the blood-based parameters, there were decreases in protein carbonylation (46%) and nitric oxide (60%) and increases in glutathione (170%) and superoxide dismutase (160%) in the depression group (*p*<0.005).

**CONCLUSIONS:**

The aquatic exercise program reduces depression and anxiety, improves functional autonomy and decreases oxidative stress in depressed elderly individuals.

## INTRODUCTION

Decreased physical activity can contribute significantly to increased levels of depression 1. On the other hand, regular physical exercise positively alters the symptoms of depression thereby promoting mental health 2,3. In addition, physical exercise facilitates and stimulates social interaction with positive consequences on quality of life 1,3. Feasible and effective interventions that might provide benefits to both physical and mental health are needed for these disorders to optimize health outcomes in this vulnerable patient group 1,4.

Recently, research in this area has been focused on mental health markers, which reflect common pathophysiological processes, related to the loss of functional autonomy and increased oxidative stress that are thought to be involved in depression 5,6,7. Psychosocial stressors, a sedentary lifestyle and low functional autonomy may alter cellular functioning and have been proposed as worthwhile intervention targets for the treatment or prevention of depression 3,4,7.

Although studies have shown significant improvement in the depressive symptoms of elderly individuals after the regular practice of physical exercises 2,8, the relations between mental health, functional autonomy and oxidative stress remain obscure. The aim of this study was to investigate the effects of aquatic exercise on mental health, functional autonomy and oxidative stress parameters in depressed elderly individuals.

## MATERIALS AND METHODS

### Ethical details

This study was approved by the National Health Council, National Research Ethics Commission resolution CNS 466/12 for research involving humans and approved by the local Ethics Committee University Extreme Sul Catarinese (CAEE 47120815.3.0000.0119).

All participants provided written consent prior to participation.

### Study design

This longitudinal clinical study, conducted for 12 weeks, subjected both the depression group and the nondepression group to the aquatic physical training program. Two days before and 48h after the program, parameters related to mental health, oxidative stress and physical fitness were analyzed.

### Group allocation

**Figure 1:**
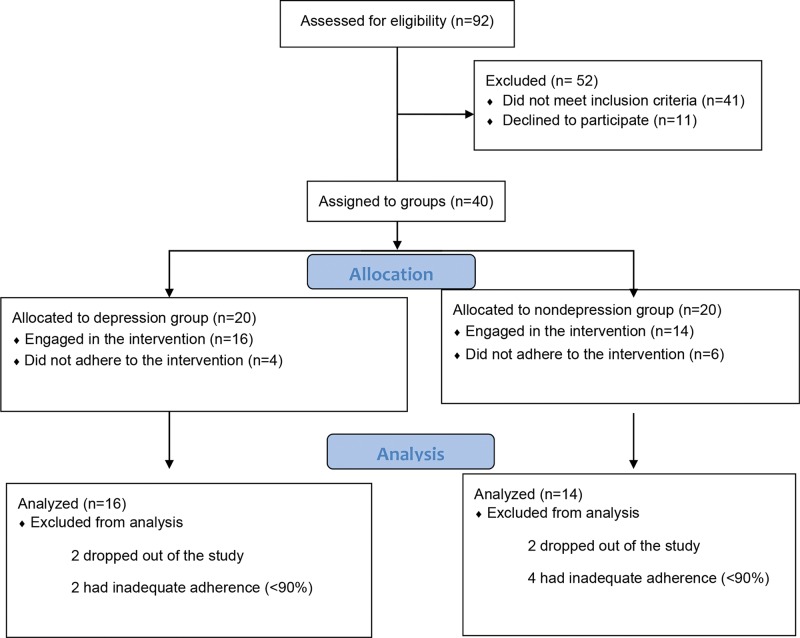
Diagram of the sample selection process for this study.

Initially, 56 patients were screened with a medical diagnosis of major depression and confirmed by the medical clinic psychiatrist at the local University, and 36 nondepressed elderly individuals participated in the study. Of the 56 patients, 29 did not meet the inclusion criteria, and 7 declined to participate. Thus, 20 patients (9 men and 11 women) with depression, aged between 50 and 80 years old, were included in the study and formed the depression group. Of the 36 nondepressed individuals, 12 did not meet the inclusion criteria, and 4 declined to participate. Thus, 20 (13 men and 7 women) elderly individuals without depression, aged between 50 and 80 years old, were included in the study and formed the nondepression group. Both groups were subjected to the same physical training program at the same place, time and days of the week. (Figure 1: allocation).

### Subjects

Patients with a diagnosis of major depressive disorder (MDD), according to established criteria (e.g., DSM5), and healthy elderly individuals (according to WHO criteria) were recruited through advertising in local newspapers. The criteria for inclusion in the depression group included the following: had MDD, were not engaged in regular exercise (i.e., did not exercise more than 20 min on 3 or more days a week), were 50 years old or over, and had a recent medical release for exercise practice (in the 6 months preceding the study). The exclusion criteria were current drug abuse, psychotherapeutic treatment, contraindications to physical exercise, suicidal behavior according to the Hamilton depression rating scale 10, and metabolic or endocrine disorders. The participants were instructed to not change the medication prescribed by a psychiatrist (Table 1) throughout the study. The inclusion criteria for the healthy elderly group were as follows: healthy elderly individuals who were without pathology or physical limitations for exercise practice, were 50 years old or over, and had a recent medical release for exercise practice (in the 6 months preceding the study). The exclusion criteria were individuals who presented with pathological problems (musculoskeletal, cardiometabolic or endocrine) that limited or were contraindicated for the practice of the programmed exercise. In addition, participants from either group who did not complete more than 90% of the stipulated physical activity program were excluded.

### Intervention

Patients underwent a 12-week aquatic training program with two weekly sessions, lasting 45 min. In both groups, the intensity was similar. Heart rate (HR) was monitored every 10 sec (Polar, Kajaani, Finland), and the rate of perceived exertion (Borg scale) was measured in the final 20 sec of each stage. The aquatic training classes were always held in the afternoon in a pool with a depth of 1.20 m, measuring 25 m×12.5 m, with water in the approximate temperature range of 26 to 28°C.

### Program exercise

Each 45-min session was divided into a warm-up period (5 min) and the main training program (40 min), followed by a cool-down period (5 min). Both groups underwent interval-training programs with the following schedule: 9 exercises in each session; each exercise was performed as 4 sets of 30 sec with 10-sec intervals; two weekly sessions (Tuesdays/Thursdays). Exercise intensity was measured by a heart rate monitor and maintained in a low-intensity range (50% and 60% of maximum HR) or a Borg scale score of 13 to 14 points. The exercises involved the large muscle groups, with upper and lower limbs together. Both groups had identical training protocols (Table 2).

### Clinical tests

These tests were conducted 48h before the first session and 48 h after the last training session in the patients for a clinical evaluation and included assessments of mental health, functional mobility, and oxidative stress. Before and after the intervention, an alimentary assessment was performed over 3 consecutive days.

### Mental health

The Beck Depression Inventory (BDI) 11 is a standardized self-administered questionnaire designed by the Center for Cognitive Therapy (CCT) researchers and is a widely used measure for the self-assessment of depression, both in research and in the clinical setting 12. The BDI is a participant-administered questionnaire with 21 items. The total score ranged from 0 to 63 points, and the items referred to sadness, pessimism, feeling of failure, lack of satisfaction and feeling guilty, among others. The Beck Anxiety Inventory (BAI) 13 presented 21 items related to anxious symptoms, and each was composed of four affirmations that increased in degree of intensity from 0 to 3. More than one affirmation could have been chosen; however, the computed score always used the affirmation of greater intensity.

### Functional mobility

For the Timed Up & Go (TUG) test, individuals were seated in a chair (45 cm high) with their back against the chair. They were instructed to stand, walk 3 m following a straight line on the ground as fast and safely as possible, return to the chair, and sit in the starting position 14. The Berg balance scale (BBS) had a maximum score of 56, and each item had an ordinal scale of 5 alternatives ranging from 0 to 4 points. The test is simple, easy to administer, and safe for the evaluation of elderly patients. These tests require only a stopwatch and a ruler as equipment, and its execution takes approximately 15 min 15. The test used to assess the flexibility of the hamstring muscles was the Sit and Reach originally proposed by Wells and Dillon in 1952, following the Canadian standardization of physical fitness assessment tests (Canadian Standardized Test of Fitness; CSTF) 16.

### Oxidative stress parameters

Protein oxidation was measured by the formation of carbonylated protein derivatives using 2.4-dinitrophenylhydrazine read spectrophotometrically at 370 nm 17. Total glutathione levels (GSH) were measured in a reaction between DTNB and thiols that reached a maximum in 5 min. Absorbance was read at 412 nm after 10 min, and a standard curve of GSH was used to calculate the GSH levels in each sample 18. Superoxide dismutase (SOD) activity was estimated by adrenaline auto-oxidation inhibition, which was read at 480 nm in a spectrophotometer 19. Nitric oxide (NO) production was estimated spectrophotometrically based on nitrite generation. Samples were incubated with Griess reagent at room temperature for 10 min, and the absorbance was read at 540 nm using a microplate reader 20.

### Blood collection

Eight-milliliter samples of blood were obtained from the antecubital vein. Blood was collected in vacutainers without additives and centrifuged at 1500 rpm for 10 min at 4°C. Aliquots of red blood cells and serum were stored at -70°C until used in the biochemical assays.

### Statistical analyses

All analyses were performed by blinded evaluation. The data are expressed as the means ± standard errors of the mean (SEM). The Kolmogorov–Smirnov test was used to confirm normality. The χ^2^ test for nonparametric analyses was also used and followed by the Bonferroni post hoc test. The a priori sample size was calculated based on a predicted difference of 0.5% in the levels of SOD=0.05 and power of 0.90, performed with IBM Statistical Package for the Social Sciences (SPSS) software (Armonk, New York; version 18), that was based on the study of Silva et al. 21. This calculation indicated that 11 patients in each group would be sufficient to detect significant changes in oxidative stress. The level of significance established for the test was *p*<0.05. SPSS version 21.0 was used as the statistical software.

## RESULTS

### Treatment groups

The study participants were divided into two groups: depression and nondepression. In the depression group, 56 patients were assessed for eligibility, 20 were allocated to the group, and 16 were included in the exercise program and analyses. The four individuals who were not included in this analysis were 2 who dropped out of the study and two who had inadequate adherence (<90%). In the nondepression group, of the 36 patients assessed for eligibility, 20 were randomized and 14 were included in the analyses and exercise program. The six individuals who were not included in this analysis included 2 who dropped out of the study and four who had inadequate adherence (<90%). To perform an intention-to-treat analysis, all participants were invited to undertake before and posttraining evaluations. The distribution of individuals can be observed in Figure 1.

### Baseline characterization of participants

Age, duration of depression, body mass, BMI, sex, and medical treatments are presented in Table 1. There were no significant differences between groups (*p*>0.05). The values are presented as the means ± SEM.

### Control of the intensity of the exercise sessions

As observed in Table 2, the results showed significant increases in HR and Borg scores in the nondepression and depression groups after 10 min (113±10 HR; 125±1 HR; 11±1 points; 12±2 points, respectively), 20 min (137±13 HR; 143±8 HR; 12±2 points; 13±1 points), 30 min (135±15 HR; 145±6 HR; 14±1 points; 14±1 points) and 40 min (128±8 HR; 131±1 HR; 13±2 points; 12±2 points) of the exercise sessions relative to before the exercise session (73±12 HR; 74±9 HR; 0 points; 0 points) (*p*<0.05).

### Depression scores

**Figure 2:**
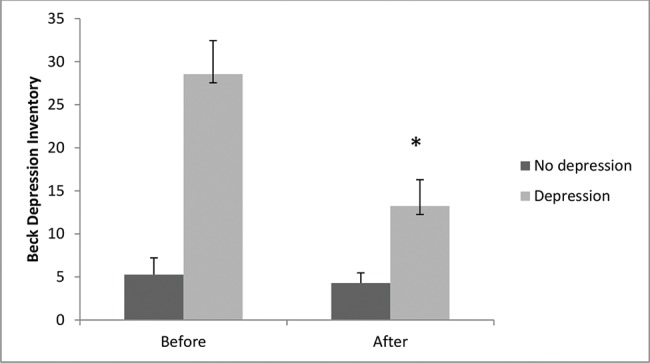
Comparison between depression levels before and after the aquatic exercise program. Data are expressed as the means ± standard errors of the mean. The χ^2^ test for nonparametric analyses was also used, followed by the Bonferroni post hoc test. The symbol (*) indicates intragroup significant differences (*p*<0.05).

The results shown in Figure 2 reveal a significant decrease in scores for depression after the aquatic exercise program in the depression group (-13.2±3 points) when compared to scores before the program (score of 28.5±3.8) (*p*<0.01). The control group did not show significantly altered results on the depression scale after (4.2±1.1) compared to before (5.2±1.9) the program (*p*>0.05). Our results indicated that clinically, there was a 53% decrease in the scores for depression in the depressed elderly individuals.

### Anxiety scores

**Figure 3:**
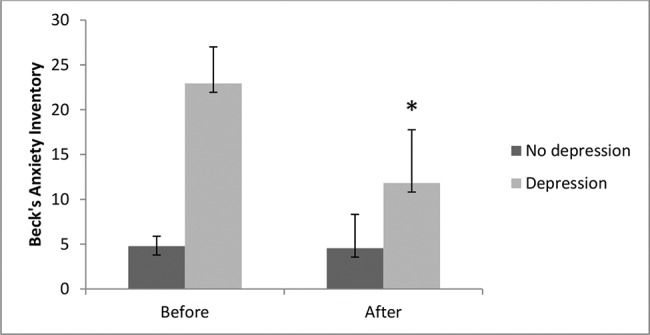
Comparison between anxiety levels before and after the aquatic exercise program. Data are expressed as the means ± standard errors of the mean. The χ^2^ test for nonparametric analyses was also used, followed by the Bonferroni post hoc test. The symbol (*) indicates intragroup significant differences (*p* <0.05).

As shown in Figure 3, the results showed a significant decrease in scores for anxiety after the aquatic exercise program in the depression group (11.8±5) when compared to before the program (22.9±4) (*p*<0.05). The control group did not show significantly altered results for anxiety after the program (4.5±3) compared to before the program (4.7±1) (*p*>0.05). Our results indicated that clinically, there was a 48% decrease in the anxiety scores in the depressed elderly individuals.

### Timed Up & Go test (TUG)

The results in Table 3 show a significant decrease in time for the Up & Go test after the aquatic exercise program in the depression group (7.68±0.3 sec) compared to before the program (11.5±0.7 sec) (*p*<0.05). The nondepression group did not have significantly altered results on the TUG test after (7.4±0.3 sec) compared to before (7.1±0.4 sec) the program (*p*>0.05). In the Berg test, the results showed a significant increase after the aquatic exercise program in the depression group (55.5±0.2) compared to before the program (50.8±1.3) (*p*<0.05). The nondepression group did not show altered results after (56±0.1) compared to before (56±0.3) the program. In addition, the results demonstrated a significant increase in the flexibility of the hamstring muscles after the aquatic exercise program in the depression group (26±2 cm) compared to before the program (18±1.8 cm) (*p*<0.05). The nondepression group did not have altered flexibility after (27±1.6 cm) compared to before (26±3.8 cm) the program. Our results indicated that clinically, there was an increase of 33% in dynamic mobility, 9% in static mobility and 44% in levels of flexibility in the depressed elderly individuals.

### Oxidative stress parameters

As observed in Table 4, the results showed a significant decrease in oxidative damage indicated by protein carbonylation after the aquatic exercise program in the depression group (0.16±0.04 nmol/mg protein) compared to before the program (0.3±0.07 nmol/mg protein) (*p*<0.01). The nondepression group did not show significantly altered oxidative damage after (0.13±0.02 nmol/mg protein) compared to before (0.14±0.03 nmol/mg protein) the program (*p*>0.05). Regarding nitric oxide, the results showed a significant decrease in its production after the aquatic exercise program in the depression group (11.7±3.5 nmol/mg protein) compared to before the program (28.2 ±2.5 nmol/mg protein) (*p*<0.001). The nondepression group had significantly increased levels of this marker after (12.8±2.5 nmol/mg protein) compared to before (5.56±2.8 nmol/mg protein) the program (*p*<0.05). Regarding superoxide dismutase, the results showed significantly increased SOD after the aquatic exercise program in the depression group (1.3±0.3 nmol/mg protein) compared to before the program (0.5±0.01 nmol/mg protein) (*p*<0.05). The nondepression group did not show altered SOD after (1.44±0.3 nmol/mg protein) compared to before (1.3±0.2 nmol/mg protein) the program (*p*>0.05). In addition, glutathione significantly increased after the aquatic exercise program in the depression group (4.17±0.8 nmol/mg protein) compared to before the program (1.54±0.07 nmol/mg protein) (*p*<0.01). The nondepression group did not show altered GSH results after (2.88±0.03 nmol/mg protein) compared to before (2.97±0.08 nmol/mg protein) the program (*p*>0.05). Our results in the depressed group indicated that clinically, there was a reduction of 46% in protein carbonylation and 60% in nitric oxide, and there was an increase of 170% in GSH and 160% in SOD.

### Dietetic parameters

Food intake, macronutrient distribution (carbohydrates, lipids and proteins) and daily calorie intake were not altered by the 12-week training period (data not shown).

## DISCUSSION

The present study demonstrates that a low-intensity aerobic training program in the aquatic environment can contribute to the treatment of depression by reducing anxiety and depression scores, improving functional autonomy and decreasing oxidative stress.

The reduction in the depression scores found in the present study demonstrated the efficacy of aquatic training on depression in elderly individuals and corroborates previous meta-analyses 7. Dunn et al. 22 randomized adults diagnosed with depression to 12 weeks of one of five aerobic exercise-training treatment conditions and demonstrated that three to five times per week presented similar results regarding the decrease in depression scores. Our results showed that the frequency of two times a week was sufficient to reduce depression. We believe that the intermittent characteristic of the exercise, unlike the traditional “continuous aerobic” method reported by previous studies 23,24, is the crucial factor in explaining these results. Several theories have been advanced to explain the antidepressant effects of exercise, including hormonal changes (e.g., increased beta-endorphins, serotonergic adaptations and hormone levels) 1,23 and oxidative stress (oxidative damage and antioxidant defense system) 25, as well as changes in cortical activity and structure 26.

In the current study, anxiety levels were reduced among the depression group; these findings were consistent with previous studies 12,27. Regular physical exercise decreased symptoms of anxiety 27. The related release of β-endorphin and dopamine induced by exercise provides a tranquilizing effect in regular practitioners 5,28. Our study demonstrated that low-intensity (Table 2) exercise was sufficient to reduce anxiety. Clinical trials have indicated that physical exercise can have antidepressant and anxiolytic effects 3. Studies involving low-intensity aquatic exercises have presented significant results for the reduction in anxiety, corroborating the present findings 28,27. Several studies have shown that during physical exercise, there is release of β-endorphin and the neurotransmitter dopamine, providing a tranquilizing and analgesic effect, thereby reducing the symptoms of anxiety 28,29.

The anatomical and physiological changes observed in the elderly population have been associated with increased disability, frailty and falls 8,30. Regarding functional autonomy, we evaluated parameters of dynamic balance (TUG), static equilibrium (Berg) and flexibility levels (Table 3). Our results point to improvements in depressed elderly people after the aquatic exercise program. Aidar et al. 8 observed similar results in which functional autonomy can be improved by 12-week aquatic physical activities. Ochoa Martinez et al. 30 demonstrated that an aquatic exercise program for 12 weeks improved the functional autonomy of older women. It is possible that the muscular resistance caused by the water 10 times higher than that of the air during the exercises required more work of the motor cortex in the elderly individuals. This improved the synchronization of the motor units and increased the excitability of the motor neurons, which reflected an increase in muscular strength and consequently the functional autonomy of the elderly individuals 31.

Depression may be accompanied by increased oxidative stress and a decreased antioxidant system 2,7. Previous studies 1,27,31 showed that oxidative damage levels were increased in persons with depression and/or depressive symptoms. Our results showed reduced oxidative damage mediated by protein carbonylation after training in the depression group (Table 4). Ji 32 suggested that the decrease in ROS production induced by physical training could increase the level of antioxidant repair of the carbonylation process. The results of this study are in accordance with the results reported by Nojima et al. 33. Both studies reinforced the idea that aerobic exercise can promote beneficial effects on oxidative damage reduction in patients with depression 2,7.

A recent study suggested the involvement of NO mechanisms in the pathogenesis of depression 34. Aggarwal et al. 34 showed that reducing NO can help improve neurobehavior related to depression-like effects in mice. Our results demonstrated decreased levels of NO after physical training in the depression group (Table 4). In humans, the regular practice of aerobic exercises increased endothelial-derived relaxation stimulated by acetylcholine by augmenting the release of NO 35.

There is also evidence to suggest that antioxidant enzymes are decreased in people with depressive symptoms 36,37. Previous studies have suggested that abnormal metabolism of SOD/GSH is closely related to various pathologies 36,37. Our results demonstrated that SOD/GSH levels were lower in the depression group and that physical training increased their activity. Several studies have reported that physical training increased SOD activity and GSH levels 32,33,38. For example, Pinho et al. 38 demonstrated in experimental models that physical training increases SOD activity in the heart. Brocardo et al. 3 showed that physical exercise increased glutathione levels in men and women. Somani et al. 39 showed that the exercise-induced increases in SOD activity and GSH content were higher than the increases in the mRNA levels of the respective antioxidants. The decrease in oxidative damage observed in the current study can be explained by the increase in antioxidants (SOD/GSH) in the depressed group.

On the other hand, there is some evidence to suggest that antidepressants have antioxidant properties and may act by reducing ROS production and improving antioxidant levels, thereby reducing oxidative stress 40,41. In our study, depressed individuals took medications throughout the exercise intervention. This is a limitation that occurs frequently for ethical reasons in studies involving humans. We believe that the use of the drugs associated with the exercise helped the treatment by improving the results of this study. Considering the reductions in anxiety and depression levels in our subjects and notable improvements in functional physical capacity and oxidative stress biomarkers after the exercise intervention, it is tempting, if not plausible, to speculate about the involvement of functional autonomy and oxidative mechanisms in obtaining beneficial responses. In conclusion, we demonstrated that an intermittent aquatic physical exercise program reduced anxiety, depression, and oxidative stress and improves the functional capacity of depressed elderly individuals.

## AUTHOR CONTRIBUTIONS

All of the authors participated in the design, interpretation of studies, data analyses and manuscript review. Silva LA, Tortelli L, Motta J, Menguer L, Mariano S, Tasca G and Silveira GB performed the experiments and were responsible for the laboratory analysis of mental health, functional autonomy and oxidative stress. Silva LA and Silveira PCL wrote the manuscript and performed the statistical analysis.

## Figures and Tables

**Table 1:** Patients’ characteristics.

	Depression	No-Depression	*p*-value
Age	59.2±7.1	58.2±8.5	0.082
Duration of depression (years)	7 (2-6)	0	
Body mass (kg)	82.9±16.1	77.1±11.1	0.091
BMI (kg^2^)	32.1±7.1	34±5	0.120
Sex (M/W)	9/7	13/7	1.000
Medical treatment			
Donarlin	3	0	
Alprozolan	4	0	
Bupopriona	2	0	
Fluoxetina	8	0	
Revotril	7	0	
Clonazepam	1	0	
Sertralina	2	0	
Venlafaxina	2	0	
Citalopram	10	0	
Brintellix	1	0	

Descriptive analysis of input data for the groups studied.

**Table 2:** Control of the intensity of the exercise sessions.

	Before	10 min	20 min	30 min	40 min	After
Nondepression (HR)	73±12	113±10*	137±13*	135±15*	128±8*	101±10
Depression	74±9	125±11*	143±8*	145±6*	131±11*	111±14
Nondepression (Borg)	0	11±1*	12±2*	14±1*	13±2*	0
Depression	0	12±2*	13±1*	14±1*	12±2*	0
Nondepression (mmHg)	121/84±10			134/83±13		135/81±9
Depression	124/76±12			138/73±16		126/79±11

Note: Values were obtained during the 1^st^, 6^th^ and 12^th^ week of the exercise program. The significant differences (*p*<0.05) are marked with (*) and indicate a difference to the before values. Heart rate (HR); Borg scale of perceived exertion (Borg); Blood pressure (mmHg).

**Table 3:** Functional mobility.

	Depression	Percent	Nondepression	*p*-value
TUG (sec)				
Before	11.5±0.7		7.4±0.3	
After	7.68±0.3*	33%	7.1±.4	0.05
BBS (scores)				
Before	50.8±1.3		56±0.3	
After	55.5±0.2*	9%	56±0.1	0.05
Flexibility (cm)				
Before	18±1.8		26±3.8	
After	26±2	44%	27±1.6	0.03

Note: Values were obtained before and after the aquatic training program. The symbol (*) indicates intragroup significant differences (*p*<0.05). Timed Up & Go (TUG); Berg balance scale (BBS).

**Table 4:** Oxidative stress parameters.

	Depression	Percent	Nondepression	*p*-value
Protein carbonylation (nmol/mg/protein)				
Before	0.30±0.07		0.14±0.03	>0.05
After	0.16±0.04^*^	46%	0.13±0.02	<0.01
Nitric oxide (umol/mg/protein)				
Before	28.2 ±2		5.56±2.8^*^	<0.05
After	11.7±3.5^*^	60%	12.8±2.5^*^	<0.001
Glutathione (umol/mg/protein)				
Before	1.54±0.07		2.97±0.08	>0.05
After	4.17±0.8^*^	170%	2.88±0.03	<0.01
Superoxide dismutase (U/mg/protein)				
Before	0.5±0.01		1.3±0.2	>0.05
After	1.3±0.3^*^	160%	1.44±0.3	<0.05

Legends: The symbol (*) indicates intragroup significant differences (*p*<0.05).
